# Working class role models in academic medicine – Professor Philip Quirke

**DOI:** 10.15694/mep.2021.000003.1

**Published:** 2021-01-11

**Authors:** Penelope Sucharitkul, Ugonna Anyamele, Mohammed Abdul Waduud

**Affiliations:** 1 Leeds Medical School; 2 Leeds Institute for Cardiovascular and Metabolic Medicine

**Keywords:** Academia, Disadvantaged, Role Models, Inspiration, Training

## Abstract

This article was migrated. The article was marked as recommended.

Representation of researchers from underprivileged backgrounds in unknown in academic medicine. We present the inspiring experiences of Professor Philip Quirke describing his humble beginnings in the East End of London to becoming an internationally acclaimed academic clinician. Importantly he offers his advice on what someone from a similar background should consider with similar aspirations.

## Can you tell us about yourself?

I was born in Romford, Essex (United Kingdom [UK]). My father was an Irish Ford factory-worker and my mother stayed at home. My parents had never taken an exam in their lives and left education at 12 years old. My siblings and I always worked on weekends; our parents really did not earn much money, but they had aspirations for us to get a good education.

I passed my 11+ exams and went to an all-boys grammar school, Coopers’ Company grammar school, in the East End of London (London, UK). When I finished my first year in sixth form, the school moved location and amalgamated with the girl’s school and then became a comprehensive school. So technically I can say I’m a comprehensive boy, but it’s a bit of a cheat because I was a “grammar boy” for 6 years. It was always a state school, but a very good state school. I did not work particularly hard at school, until my chemistry teacher scolded me so I started working hard during the last 3 months of my A Levels. I just scraped into Southampton medical school with two Bs and a C.

## When did you realise you wanted to pursue academic medicine?

During my A-levels, I was on a Zoology field trip surveying moss when I noticed something unusual under the microscope. Out of sheer curiosity, I took it to my professor who sent it for further investigation. I then received a letter saying that it was actually a new species! So at 17 years old, serendipity, luck and amazement in discovering something new, played a massive role in my career. That was my first academic experience. Curiosity led me to publish three further case reports of rare conditions during medical school, which have served fantastically on my CV.

## What is your current role, specifically in academic medicine?

I have various titles. I am Professor of Pathology at the University of Leeds and Head of Leeds Institute of Medical Research Division of Pathology and Data Analytics. I am also a National Institute for Health Research Senior Investigator and Fellow of the Academy of Medical Science. My main research interest is in bowel cancer, which involves international collaboration.

I am also an Honorary Consultant Histopathologist in the National Health Service (NHS) and Lead for the Pathology Colorectal Cancer Group. I also chair the NHS Bowel Screening Programme Pathology Committee. In addition, I am the immediate past president of the Pathological Society of Great Britain and Ireland. Furthermore, I am the Academic Training Programme Director for Health Education England for Leeds West (UK).

I only do things that are fun! There’s no point doing something you don’t enjoy. That is one big benefit of being an academic. You are able to choose what you want to do instead of doing the same things every day for the rest of your life.

## Did you encounter many people from similar backgrounds to yourself throughout your career?

In terms of white working-class individuals, there was my friend Bob, who was also from London (UK), but there were relatively few of us. It wasn’t really an issue - people were just people. We weren’t as confident as the students from public schools but they tended to drop out more because they couldn’t be bothered to work. There are a lot of advantages to being working class. It builds your resilience. For instance, Saturday work was a good experience. However, I spent all my time working instead of doing school work, so I didn’t do as well in school. Although I don’t think this particularly held me back - I accommodated.

There were some, but not many, people from Black And Minority Ethnic (BAME) backgrounds. There was one individual who stood out, Dr Chai Patel, who was from a middle-class background. He went on to run the Priory group after leaving medicine in Oxford (UK). He was super intelligent, and he worked hard. He was very personable, but he didn’t progress in medicine as he was not given the opportunities he deserved. Anyhow, he’s shown how good he was by going on to becoming really successful. He was a real loss to medicine.

## Do you think that academics from disadvantaged backgrounds are well represented in academic medicine?

No, we are not. Although, I didn’t realise how disadvantaged I was at the time. However, it’s not something that people seemed to openly discuss in the past, and even now. You won’t know until you begin those conversations - you get surprised by some of the people who are from disadvantaged backgrounds, as you don’t seem to expect it. I think we should be more open to these conversations.

## Who were your role models in academic medicine?

Many of my role models in academic medicine were Professors in Pathology, as well as Sir David Barker who was a Professor of Medicine. He organised my elective in Tanzania. Most had senior positions such as Deans, and Heads of Department.

## Did coming from a disadvantaged background present any obstacles in your academic journey?

Someone is disadvantaged because they lack the knowledge, they lack the contacts, and they lack the expectations. My parents were always supporters of education, but not until later did they express that we should go to university. They would never have thought that my brothers and I could have gone to university; the fact that I became a doctor was astounding for my mum. I was probably disadvantaged because I didn’t know any doctors, medical students or even anyone with a degree! I knew hardly anything about getting into medical school except that you needed good grades. There were virtually no opportunities. I had to form those networks from scratch and look for opportunities myself, which took initiative and self-motivation. Whereas, if you’ve been sent to public school and receive the best teaching and opportunities and are expected to walk out with top grades, then you may not have developed the drive to motivate yourself at university. At university the only driver is you. I think there are big advantages if you manage to overcome obstacles in your life and turn them into positive experiences. This makes you more resilient. You mustn’t ever feel a victim, because you would bear that like a weight for the rest of your life. The victims are the people who don’t make it.

## Did you receive any financial support (i.e. funding, scholarships) to help you throughout your career based on your background?

When I left school, I was given an “exhibition” from the Coopers Company. This was back in 1974, £50 per year for four years, which paid for a part of the costs to purchase my books. That money went a long way back then. I also didn’t have to pay fees and had a means-tested grant, so my parents contributed a small bit of money - but there was no formal financial support at medical school.

Nowadays, there’s more financial support available for medical students from disadvantaged backgrounds through universities. It is a matter of thoroughly researching the scholarships and support available at that specific university. In terms of academic medicine, I would advise seeking a mentor, though it may be difficult to find mentors who are from similar, disadvantaged or minority backgrounds.

During my undergraduate education, intercalated degrees were not available; instead, everybody did a 4 ^th^ year research project in medical school, as this was part of the course. So, I managed to obtain research experience from that. I think intercalated degrees these days can be discriminatory, because finance can be a problem for some students, as it is an extra year of student finance.

## What are your tips for student from disadvantaged backgrounds looking at pursuing a career in academic medicine?

Always ask questions, especially if something does not make sense. Make a list of resources, such as financial support, that you are eligible for. You don’t know what is out there until you look and give it a go. There are some really surprising opportunities.

Join academic committees, such as the National Student Association of Medical Research (NSAMR, UK) student body and get involved. Aim to be a well-rounded person with a range of transferrable skills, for instance through sport or volunteering. Seek a mentor for support. Finally, you need to learn that in academic medicine, the rewards come later. You’ve got to be patient, work hard and embark on this journey as positively as you can because obstacles are an inherent part of life. Instead of feeling restrained by circumstances, make the most out of every opportunity and make a determined effort to work towards your goal.

## Take Home Messages

• Your social background does not necessarily limit your future success in academic medicine.
• Embrace challanges that present during your academic career.
• Make yourself aware and take advantage of opportunities which can boost your skills, broaden your prospects and widen your experience.

## Notes On Contributors

**Penelope Sucharitkul** is an intercalating medical student at the University of Leeds, UK. She is the only child from a single-parent family, and currently lives with her father who is a Factory Worker. She had a disrupted primary school education, however later went onto study in a selective state grammar school in Essex, UK.

**Ugonna Anyamele** is a third year medical student at the University of Leeds, UK. She attended a comprehensive secondary school in Lambeth, London, and has been involved in running events as part of the Widening Access to Medical School scheme. She recently completed an academic summer internship funded by MedTech Foundation (University of Leeds, UK).

**Mohammed Abdul Waduud** is a British Heart Foundation Research Fellow in Vascular Surgery working at the University of Leeds, UK. He attended a state secondary school in one of London’s (UK) most deprived Boroughs, Tower Hamlets, and later attained a scholarship to study at Westminster School.

## Declarations

The author has declared that there are no conflicts of interest.

## Ethics Statement

Ethics approval was not required for this article as this was an interview style case study. Professor Philip Quirke agreed to the interview provided it would be written up for publication to share the valuable content.

## External Funding

This article has not had any External Funding

