# Supplementary material for: Working class role models in academic medicine – Professor Philip Quirke
Source: MedEdPublish (2016). 2021 Jan 11;10:3. [Version 1] doi: 10.15694/mep.2021.000003.1 (PMC10939597; doi:10.15694/mep.2021.000003.1)
Supplement: Peer review history

# Reviewer report: Reviewer response for version 1

**DOI:** 10.21956/mep.18931.r27072

**Published:** 2021-05-10

**Authors:** Madalena Folque Patricio

**Affiliations:** 1 School of Medicine, University of Lisbon

This review has been migrated. The reviewer awarded 5 stars out of 5 It was with great interest that I read this interview.Congratulations to the authors for drawing the readers’ attention to some aspects that deserve to be highlighted:• Positive family expectations, despite the unfavourable context and their own life story• The importance of ‘specific moments’ in life, which cannot go unnoticed• The importance of self-determination and internal motivation for the successful journey • The additional contribution of external motivations • The fact that ‘not everything in life is pre-determined’• The fact that this narrative per se is a very good ‘role model’If I may suggest something, I would have liked to hear it in moments of failure, moments of disappointment, moments when things were not going well, because those moments must have happened. The objective is to hear how to overcome these difficult periods, how it was possible to find the strength to overcome them, what was the support for resilience.This inspirational journey has not always been a fairy tale, so as a ´role model’, it would be important to identify how resilience has managed to overcome vulnerability. This will be the great lesson we can learn from this interview.A final suggestion is to share this interview with IFMSA, EMSA and other medical students' associations to have other students inspired by Professor Philip Quirke.

Reviewer Expertise:

NA

No decision status is available

# Reviewer report: Reviewer response for version 1

**DOI:** 10.21956/mep.18931.r27071

**Published:** 2021-03-30

**Authors:** Ken Masters

**Affiliations:** 1 Sultan Qaboos University

This review has been migrated. The reviewer awarded 5 stars out of 5 An interesting interview with Professor Philip Quirke as an example of a working-class role model in academic medicine.The story of the moss is both fascinating, and a valuable lesson for educators, as it is illustrative of the types of things that are required for a spark: curiosity, support, affirmation, credit. These motivators, both internal and external, are crucial at that age (probably at all ages), and can have a lasting impact on a young person’s life: a valuable lesson for all teachers. (One could begin a teaching workshop with this paragraph).It is an inspiring story, indeed, and also well-balanced: important from the paper is the message from Dr. Patel’s experience. Unfortunately, although, it is possible for hard work and effort to be recognised and rewarded, this does not always happen. The result is a loss for everyone.Finally, although the specific circumstances will differ from situation to situation, the advice given will be useful for all. Particularly that which alerts people to the fact that there is often assistance available, but it is not always well-advertised, and one might not be aware that it exists, so it is worth investigating.A good and inspiring read. Thank you.

Reviewer Expertise:

NA

No decision status is available

# Reviewer report: Reviewer response for version 1

**DOI:** 10.21956/mep.18931.r27073

**Published:** 2021-01-28

**Authors:** Annwyne Houldsworth

**Affiliations:** 1 HECL

This review has been migrated. The reviewer awarded 5 stars out of 5 Thank you so much for presenting this very inspiring narrative about Prof Philip Quirke.It is clear that socioeconomic factors are frequently important factors in many life outcomes, poverty being one of those factors. It is very refreshing to read about someone who has escaped many of the constraints associated with social and family background.The Coleman report says, ‘‘the inequalities imposed on children by their home, neighbourhood and peer environment are carried along to become the inequalities with which they confront adult life at the end of school”. The nature and strength of the Prof Quirke’s strength of character is a hugely inspiring example for many prospective students. The professor describes his parents’ anticipated destiny for him not to progress academically, despite their formidable attempts to encourage and support their son’s education.It is clear that education sometimes reproduces the inequalities of socioeconomic status but this is not necessarily due to the quality of the teaching and learning in the school as this remains high in the UK state schools but self confidence and esteem are important in private education, often exceeding natural intellectual capacity.The comparison of funding between 1970’s and today was extremely interesting, having supported medical students applying for extra funding; I understand the difficulties involved with this.Some academic theoretical references about this subject would enhance the text and give academic context to this opinion piece.The advice to seek a mentor is a very valuable suggestion for medical students. It’s a bit late but could Prof Quirke be my mentor?

Reviewer Expertise:

NA

No decision status is available
